# Computational and mitochondrial functional studies of novel compound heterozygous variants in *SPATA5* gene support a causal link with epileptogenic encephalopathy

**DOI:** 10.1186/s40246-023-00463-x

**Published:** 2023-02-27

**Authors:** Víctor Raggio, Martín Graña, Erik Winiarski, Santiago Mansilla, Camila Simoes, Soledad Rodríguez, Mariana Brandes, Alejandra Tapié, Laura Rodríguez, Lucía Cibils, Martina Alonso, Jennyfer Martínez, Tamara Fernández-Calero, Fernanda Domínguez, Melania Rosas Mezquida, Laura Castro, Alfredo Cerisola, Hugo Naya, Adriana Cassina, Celia Quijano, Lucía Spangenberg

**Affiliations:** 1 Departamento de Genética, Facultad de Medicina, Universidad de la República, Montevideo, Uruguay; 2 Bioinformatics Unit, Institut Pasteur de Montevideo, Montevideo, Uruguay; 3 Departamento de Histología y Embriología, Facultad de Medicina, Universidad de la República, Montevideo, Uruguay; 4 Departamento de Métodos Cuantitativos, Facultad de Medicina, Universidad de la República, Montevideo, Uruguay; 5 Centro de Investigaciones Biomédicas (CEINBIO), Universidad de la República, Montevideo, Uruguay; 6 Departamento Básico de Medicina, Hospital de Clínicas, Facultad de Medicina, Universidad de la República, Montevideo, Uruguay; 7 Departamento de Neuropediatría, Facultad de Medicina, Universidad de la República, Montevideo, Uruguay; 8 Departamento de Bioquímica, Facultad de Medicina, Universidad de la República, Montevideo, Uruguay; 9 Department of Exact and Natural Sciences, Universidad Católica del Uruguay, 11600 Montevideo, Uruguay; 10 Universidad Católica del Uruguay, 11600 Montevideo, Uruguay; 11 Departamento de Producción Animal y Pasturas, Facultad de Agronomía, Universidad de la República, Montevideo, Uruguay

## Abstract

The *SPATA5* gene encodes a 892 amino-acids long protein that has a putative mitochondrial targeting sequence and has been proposed to function in maintenance of mitochondrial function and integrity during mouse spermatogenesis. Several studies have associated homozygous or compound heterozygous mutations in *SPATA5* gene to microcephaly, intellectual disability, seizures and hearing loss. This suggests a role of the *SPATA5* gene also in neuronal development. Recently, our group presented results validating the use of blood cells for the assessment of mitochondrial function for diagnosis and follow-up of mitochondrial disease, minimizing the need for invasive procedures such as muscle biopsy. In this study, we were able to diagnose a patient with epileptogenic encephalopathy using next generation sequencing. We found two novel compound heterozygous variants in *SPATA5* that are most likely causative. To analyze the impact of *SPATA5* mutations on mitochondrial functional studies directly on the patients' mononuclear cells and platelets were undertaken. Oxygen consumption rates in platelets and PBMCs were impaired in the patient when compared to a healthy control. Also, a decrease in mitochondrial mass was observed in the patient monocytes with respect to the control. This suggests a true pathogenic effect of the mutations in mitochondrial function, especially in energy production and possibly biogenesis, leading to the observed phenotype.

**Supplementary Information:**

The online version contains supplementary material available at 10.1186/s40246-023-00463-x.

## Introduction

The *SPATA5* gene (MIM: 613940) encodes a 892 amino-acids long protein (spermatogenesis-associated protein 5) that is a member of the AAA (ATPase associated with diverse activities) subfamily. This protein has a putative mitochondrial targeting sequence and has been proposed to function in maintenance of mitochondrial function and integrity during mouse spermatogenesis. It has ubiquitous tissue expression, including brain, testes, spleen, intestines, among others [1].

Several studies have associated homozygous or compound heterozygous mutations in *SPATA5* gene to microcephaly, intellectual disability, seizures and hearing loss (MIM: 616577) [2–5]. This suggests a role of the *SPATA5* gene not only in spermatogenesis, but also in neuronal development [5].

Puusepp and collaborators have reviewed all (25) patients with *SPATA5* mutations identified so far and characterized the impact of silencing *SPATA5* in mitochondrial dynamics. Imbalance in mitochondrial fusion–fission rates, impaired energy production and shortening of axons were shown to occur when *SPATA5* levels are reduced [5]. This evidence elucidates some of the mitochondrial pathophysiological mechanisms in the context of *SPATA5* downregulation.

Braun et al. [6] have recently undertaken a comprehensive workup, including muscle proteomic profiling and microscopic studies to further characterize the disease in a patient with compound heterozygous mutations in *SPATA5*. This study found not only a dysregulation of mitochondrial proteins but also of proteins acting elsewhere, showing a broader complexity of this phenotype.

Recently, our group presented results validating the use of blood cells for the assessment of mitochondrial function for diagnosis and follow-up of mitochondrial disease, minimizing the need for invasive procedures such as muscle biopsies [7]. In this study, we were able to diagnose a patient with epileptogenic encephalopathy using NGS. We found two novel compound heterozygous variants in *SPATA5* that are most likely causative. To analyze the impact of *SPATA5* mutations on mitochondrial function studies on the patients' peripheral blood mononuclear cells (PBMCs), monocytes and platelets were undertaken. Oxygen consumption rates in PBMCs and platelets were impaired in the patient when compared to a healthy control. Also, a deficit in mitochondrial mass was observed in monocytes of the patient with respect to the control. This suggests a true pathogenic effect of the mutations in mitochondrial function, especially in energy production, and possibly mitochondrial mass, leading to the observed phenotype. Our results also provide additional support for the use of blood cells for assessing mitochondrial functions.

## Methods

### Sequencing and bioinformatics analysis

Genomic DNA was extracted from 100 μl of whole blood using Qiamp® DNA Blood Mini kit (Qiagen, Germany) according to the manufacturer’s instructions. We did a whole genome sequencing of the patient with 30X in a Hiseq X ten Illumina sequencing machine. FastQC [8, 9] was used for the quality of reads, and BWA [10] for read mapping onto the human genome (GRCh37). Variant calling was undertaken using GATK [11] (best practices). ANNOVAR [12] was used then for the annotation. Different sets of filters were used in order to detect potentially causative mutations (see “Variants filtering scheme” Section).

One of the candidate mutations (the frameshift) was further evaluated with the SIFT Indel tool [13], to characterize its pathogenicity effect. Additionally, the mitochondrial genome was analyzed using MToolBox [14]. The MToolBox pipeline allows the extraction of mtDNA data from WES and WGS. In order to remove contaminating nuclear-integrated mitochondrial sequences and amplification artifacts, the input reads mapped on mtDNA are realigned onto the nuclear genome (hg19/GRCh37), previous to INDEL realignment and PCR duplicates removal.

Sanger sequencing of the region of the candidate variant y *SPATA5* gene was performed on the patient, his mother and father, according to Macrogen’s pipeline. Sequencer: ABI PRISM 3730XL Analyzer and Variant analysis: Variant Reporter Software Version 2.1 (Applied Biosystems), DNASTAR Lasergene SeqMan 7.0 and Macrogen SNP analysis program v1.0.

### Variants filtering scheme

In order to filter and prioritize the variants found, we used the following rationale:

1. Homozygous mutations in coding/splicing region with a population frequency lower than 1%.
2. Heterozygous mutations in coding/splicing region with at least two variants in the same gene and a population frequency lower than 1% (compound heterozygous).
3. Heterozygous mutations in coding/splicing region with a population frequency less than 0.5%.
4. Mitochondrial mutations with high heteroplasmy (> 10%) and in coding regions or tRNA or rRNA genes (and not part of the definition of the haplogroup and not in D-LOOP).
5. Non-coding variants, either with “uncertain significance” (VUS) or “pathogenic/Likely pathogenic” or “conflicting interpretations of pathogenicity” classifications, as determined by ClinVar [15].

Of note, by splicing site mutations we consider mutations falling within the canonical donor (GT) or acceptor (AG) sites.

### Mitochondrial mass and morphology

Blood samples from the patient and control individual were freshly processed. PBMCs were isolated by density gradient centrifugation using Lymphoprep™ (Stemcell Technologies), according to the manufacturer’s instructions. Human blood monocytes were then obtained by negative selection, using antibody-coated magnetic microbeads, following the manufacturer's protocol (Pan Monocyte Isolation kit, Milteny Biotec™).

The monocytes obtained were stained with MitoTracker Green (Invitrogen™) at 15 nM for mitochondrial network visualization and Hoescht (Sigma Aldrich™) at 0.1 mg/ml for nuclei stain.

Using confocal microscopy (LeicaTCS SP5 II; Leica Microsystems, Wetzlar, Germany), we acquired images of the mitochondrial network. For the analysis of the mitochondrial mass, we applied a mask on each image and selected the regions of interest (ROIs) in FIJI free software [16] and all images were deconvolved and quantified with Huygens Professional Software (Scientific Volume Imaging, The Netherlands, http://svi.nl). For the analysis of mitochondrial morphology, we used the plugin MiNa—mitochondrial network analysis of the FIJI free software. We obtained the skeleton of the mitochondrial network stained with MitoTracker green of all the images and determined the number of mitochondria with puncta, filament and network morphology.

### Oxygen consumption rate analysis

Platelet and PBMCs isolation and oxygen consumption rate (OCR) analyses were performed as previously described [7]. Briefly, blood samples were obtained in BD Vacutainer tubes containing EDTA after an overnight fast. Platelets were isolated by centrifugation, while PBMCs were isolated by density gradient centrifugation using Lymphoprep™ (Stemcell Technologies), according to the manufacturer’s instructions. Platelet and PBMCs concentrations were determined using the automated cell counter Z1 Coulter Particle Counter (Beckman).

Oxygen consumption rate (OCR) analyses were performed in a Seahorse XFe24 extracellular flux analyzer (Agilent). Purified platelets were seeded (2.5 × 10^7^ cells/well) in 100 µl of Seahorse medium (8.3 g/L DMEM, 1.85 g/L NaCl, 5 mM glucose, 1 mM pyruvate, 2 mM glutamine, 5 mM HEPES, pH 7.4) on XFe24 V7 cell culture plates (Agilent), and the plates centrifuged at 300 g for 10 min to attach the platelets to the bottom of the plate. PBMCs were seeded (4 × 10^5^ cells/well in 100 µl of Seahorse medium) on poly-D-lysine coated XFe24 V7cell culture plates and incubated for 30 min at 37 °C to allow the adhesion to the plate. Seahorse medium (500 µl) was added to each well; plates were kept at 37 °C for approximately 1 h and loaded into the instrument.

Oxygen consumption rate was measured before and after the sequential addition of 2.5 µM oligomycin (ATP synthase inhibitor), cyanide p- (trifluoro-methoxy) phenyl-hydrazone (FCCP, uncoupler 0.5–3 µM) and 2.5/2.5 µM antimycin A/rotenone (complex III and I inhibitors, respectively). The non-mitochondrial oxygen consumption rate (obtained after the addition of antimycin A/rotenone) was subtracted from all measurements. Respiratory parameters were obtained as follows: basal (baseline OCR); ATP-independent (OCR resistant to the addition of oligomycin, proton leak); ATP-dependent (basal—ATP-independent); maximum (OCR obtained after the addition of FCCP); spare respiratory capacity (maximum—basal) [17]. Respiration was normalized considering cell number.

### mtDNA copy number per cell

DNA was extracted from PBMCs using QIAamp® DNA Mini Kit (QIAGEN), and the concentration was determined in a QubitTM 4 fluorometer (Invitrogen) using Qubit dsDNA assay kits. The number of copies/μL of mtDNA and nDNA was determined by digital droplet PCR (ddPCR) in a QX200 Droplet Digital™ PCR System (Bio-Rad), using primers targeting a region (Additional file 1: Table S1) of the nuclear gene β2M (a single-copy nuclear locus) and a region located in the minor arc of mtDNA (mtMinArc), where large deletions are rare [18].

The number of mtDNA copies per cell was determined considering the ratio of mtDNA/nDNA and that there are two copies per cell of the β2M gene [18], which means:

$${\text{mtDNA copies}}/{\text{cell}}\, = \,\left( {{2}\, \times \,{\text{mtDNA}}} \right)/{\text{nDNA}}$$

### Protein structural analyses

Using *SPATA5* canonical isoform 1 (https://www.uniprot.org/uniprot/Q8NB90), we used evolutionary information to assess whether and how protein structure is affected by our mutations of interest, namely, the missense mutation (p.G667fs; V766M) and frameshift deletion (NM_001317799:exon11:c.1999delG:p.G667fs,NM_001345856:exon11:c.1999delG:p.G667fs, NM_145207:exon11:c.2002delG:p.G668fs). Strict conservation or prevalence of residues with similar physicochemical properties (i.e., hydrophobic) in homologous proteins was considered proxies for functional/structural relevance of the missense mutation. We did this through HMMER [19] searches (default parameters) against eukaryotic proteins within UniprotKB [20], as well as Jackhmmer [21] searches (2 iterations) restricted to mammalian reference proteomes, using more stringent criteria (“–pextend 0.1 –popen 0.005” options). For mammalian homologs, we also examined a restricted sequence group sharing with *SPATA5* the exact Pfam [22] domain architecture, i.e., two ATPase domains (AAA+, Pfam PF00004), intercalated with two ATPase lid domains (AAA_lid_3, PF17862), together composing the C-terminal region starting at position 390. We aligned full-length sequences of this group with MAFFT [23] (“L-INS-i option”), including in-paralogs and alternative isoforms. The alignment was manually inspected after removing columns with more than 90% gaps using TrimAl [24]. Searching similar sequences on the Protein Data Bank (PDB) [25] returned hits from canonical ATPases as the only relevant matches. With no available *SPATA5* experimental structure, we turned to deep-learning methods, which recently showed an unparalleled success in accurate protein structure prediction [26]. Thus, an AlphaFold (AF) model for *SPATA5* main isoform served to assess mutational impacts on structure and function. For structural comparisons, we relied on experimental data for *DRG1* –*SPATA5* homolog in yeast, whose structure was recently solved by Cryo-EM [27]. In such comparisons, high confidence regions of the AF model helped to define domain boundaries and consequently reliable alignments. We proceeded as follows for structural comparisons: a *SPATA5* AF model was energy-minimized using the YASARA force field [28] and next superposed to a single chain from yeast’s *DRG1* homohexamer (PDB code 7KNU), using DALI [29], SSM [30] and CE [31]. Deriving structural alignments from various algorithms stems from the well-known difficulty of finding solutions that are both optimal and biologically meaningful [32].

## Results

### Case report

Here, we report the case of a 10 years old boy with a developmental and epileptic encephalopathy with severe global developmental delay and epilepsy. His parents are healthy and non-consanguineous. He has no siblings and other family members are healthy. Pregnancy and delivery by cesarean section (due to arrested labor) at term were uneventful. No intrauterine growth restriction or other adverse perinatal outcomes were present.

Seizures began when he was 3 months old. From 6 months of age, neurodevelopmental regression was observed, with loss of gaze contact and bubbling. Hypsarrhythmia was detected on an EEG when he was 10 months old, without clinical spasms. Seizure types included tonic seizures, tonic–clonic seizures, myoclonic seizures and epileptic spasms. Different antiepileptic drugs were administered but seizures remain refractory, currently being under treatment with lamotrigine and cannabis oil, with atonic and myoclonic seizures. Serial EEGs showed a slow and disorganized background rhythm, with bursts of brief slow generalized spikes and polyspikes associated with multifocal epileptiform activity, without posterior predominance. Photic intermittent stimulation at 10 years of age did not provoke any response.

Head circumference showed a progressive reduced growth over time, being actually a microcephaly with a cranial circumference of 49 cm (− 3 standard deviation SD). No dysmorphic features were present. Lack of visual fixation and tracking were noticed since he was 6 months old. Afterward, neither verbal nor non-verbal language or social interaction developed. Hyperreflexia, global hypotonia with repetitive, purposeless hand movements similar to stereotypes and distal athetoid movement were observed.

**Fig. 1:**
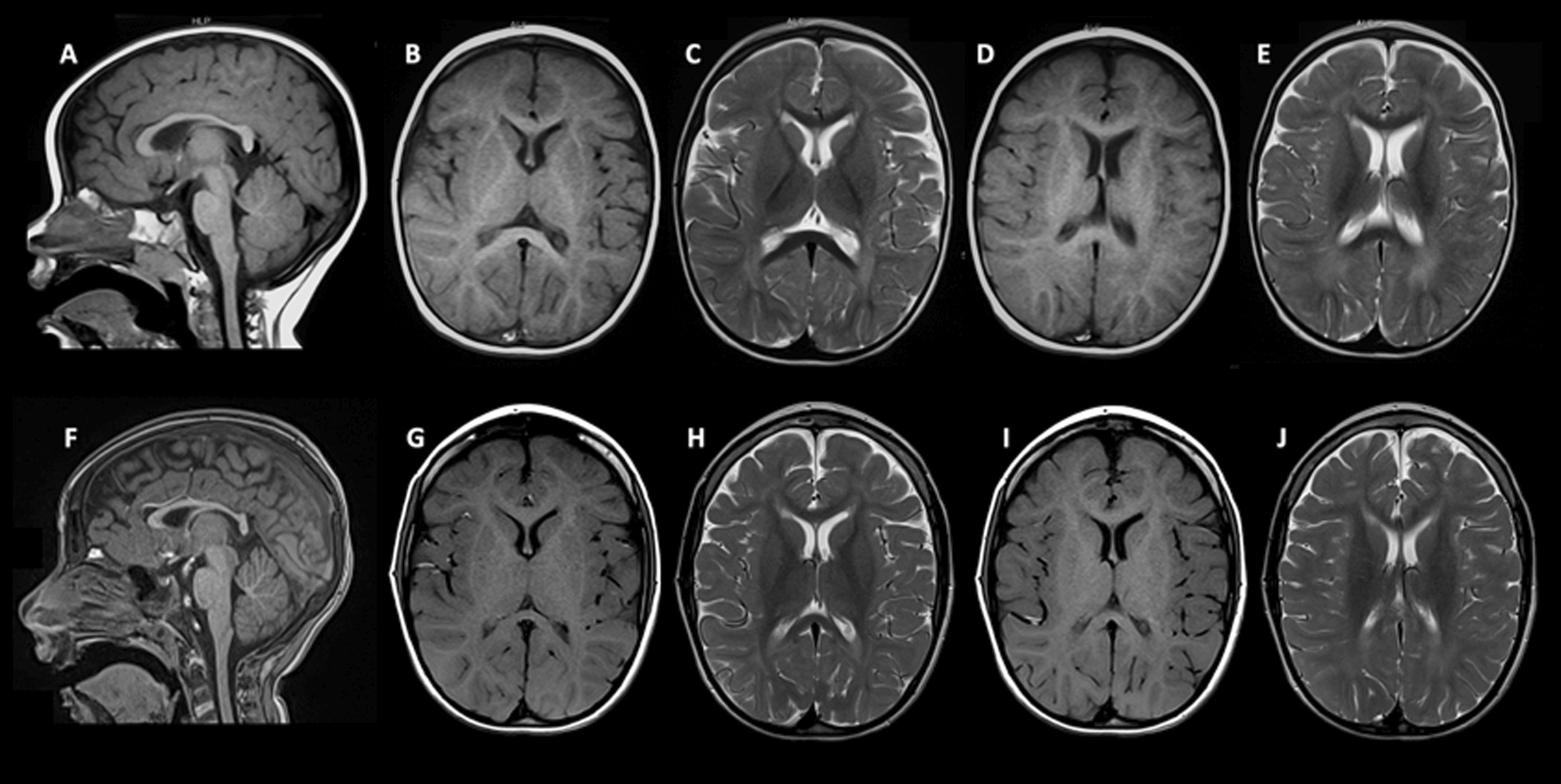
MRI at 14 months of age (**A**–**E**) and at 7 years of age (**F**–**J**). Thin corpus callosum was observed on sagital T1 sequence (**A**, **F**). Slight bilateral hypomyelination with hypointense T1 signal (**B**, **D**, **G**, **I**) and hyperintense T2 signal (**C**, **E**, **H**, **J**) in frontal and occipito-parietal periventricular and deep white matter, was observed

Cranial MRIs showed thin corpus callosum with slight bilateral hypomyelination with hypointense T1 signal and hyperintense T2 signal in frontal and occipito-parietal periventricular and deep white matter (Fig. 1A–J) Spectroscopy was normal. No progressive brain atrophy was observed. Visual evoked potential configuration and amplitude were normal. Initial auditory evoked potentials were normal but, afterward, moderate hearing loss developed with 50 dB of auditory stimulus threshold.

Echocardiogram was normal. Blood count, plasma amino-acids, biotinidase and acylcarnitines profile and urinary organic acids, were all normal. Cerebrospinal fluid (CSF) analysis and CSF amino-acids were normal. There is no history of repeated infections or hematological problems.

Previous studies in search of a genetic etiology were as follows: karyotype was normal for a male: 46,XY. Sequencing of ARX and MECP2 genes showed no pathogenic variants. No microarray was performed.

### Compound heterozygous variants with pathogenicity evidence found in SPATA5

The whole genome sequencing (WGS) of the patient revealed a total of 4,845,490 variants after bioinformatics pipeline (methods 2.1). After the proposed filtering scheme (Methods 2.2), we obtained 18 homozygous or hemizygous variants with population frequency less than 1% located at splicing sites or coding regions, 467 heterozygous variants with at least two variants in the same gene with population frequency less than 1%, 401 heterozygous variants with population frequency less than 0.5% located at splicing sites or coding regions, 33 mitochondrial variants with high heteroplasmy (> 10%) located in coding regions or tRNA, and no variants in non-coding regions with low frequency considered Likely pathogenic/pathogenic or with conflicting interpretations of pathogenicity.

**Fig. 2:**
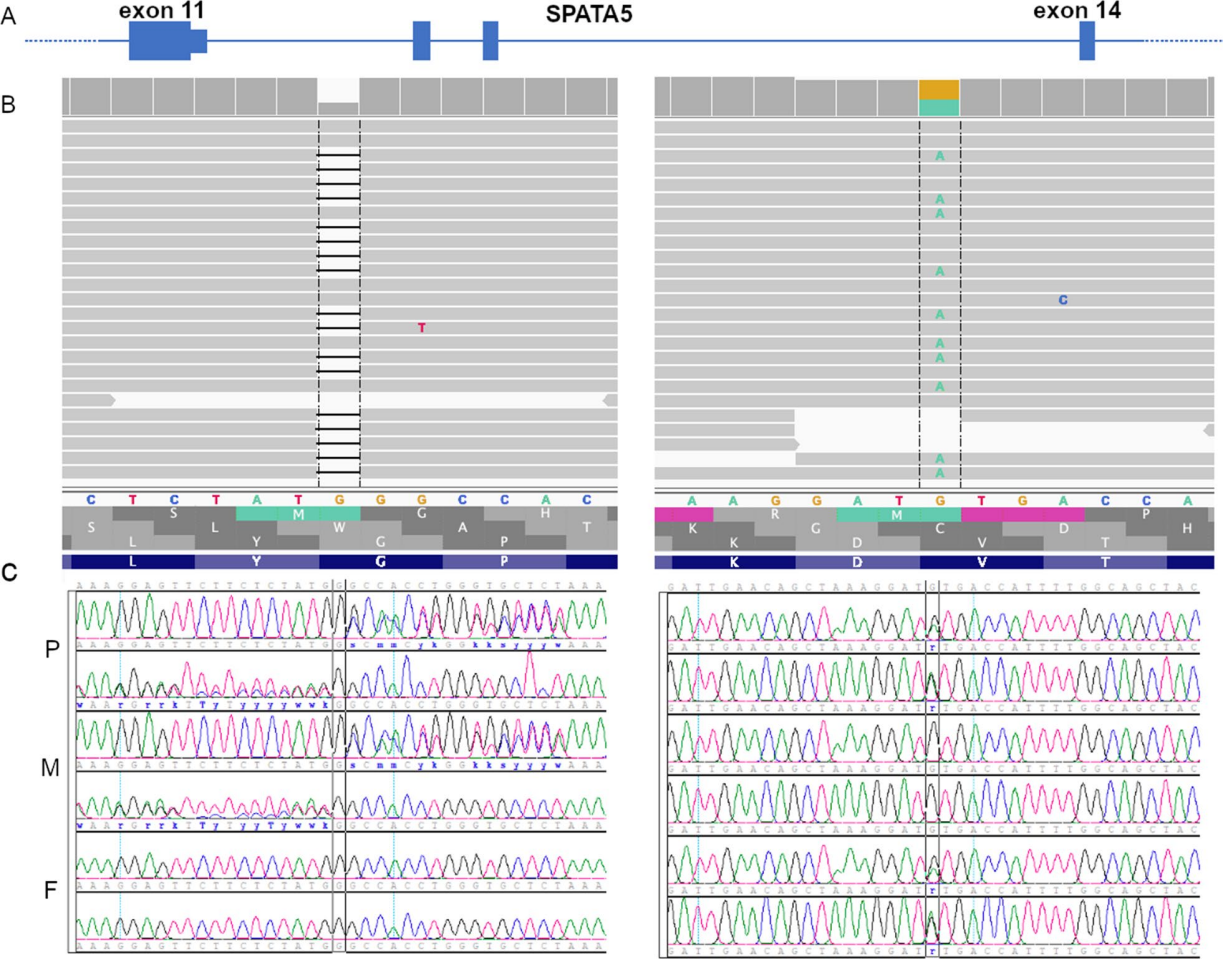
NGS and Sanger outputs (IGV and electropherogram) sequencing results. **A** Schematic SPATA5 gene partial structure. Only exons 11, 12, 13 and 14 are shown. **B** Patients’ NGS results in both relevant regions. To the left, the frameshift deletion, to the right the missense variant. **C** Sanger sequencing results on the patient (P), mother (M) and father (F) showing that the frameshift deletion is inherited from the mother (left) and the missense variant is inherited from the father (right)

Two novel heterozygous variants, in *trans* configurations, were identified in the *SPATA5* gene (Fig. 2A, B): (i) a frameshift deletion, chr4:123949473-123949473, NM_001317799: exon 11: c.1999delG, p.G667fs with no population frequency reported. The deletion lies approximately in the 75% of the protein, causes a stop-codon after nine residues from the change and it is predicted to be damaging and activate a mechanism of nonsense-mediated decay (NMD) according to SIFT [13]. (ii) A missense variant, chr4:124011816-124011816, NM_001345856:exon14:c.G2293A, p.V766M, with no population frequency reported, with a deleterious classification of in silico scores Sift, Polyphen, LRT and MutationTaster. Figure 2B shows the IGV view of the frameshift (left panel) and the missense (right panel), both having a good coverage of 29× and 22×, respectively. Sanger sequencing on mother and father revealed that mother carries the frameshift and father the missense (Fig. 2C, left and right panel). Additionally, the mutation falls in the AAA2 domain which is the most functionally relevant domain for the protein [5]. It defines the beginning of a beta strand connected to an alpha helix through a loop. According to evolutionary analysis it is a highly conserved residue and region in all vertebrates. Indeed, aligning all mammalian protein sequences having the same Pfam domains as *SPATA5*, we found the position has 94.2% valine and 3.6% isoleucine (2.2% gaps), both small hydrophobic residues. The change from valine (V) to methionine (M) would not alter the hydrophobicity (M is also hydrophobic), yet the bigger volume of M (131 Dalton in comparison to the 99 Dalton of valine) is expected to distort the protein structure, precisely in the connection of a beta strand and a loop.

### Mitochondrial mass deficit in the patient with respect to a healthy control

**Fig. 3:**
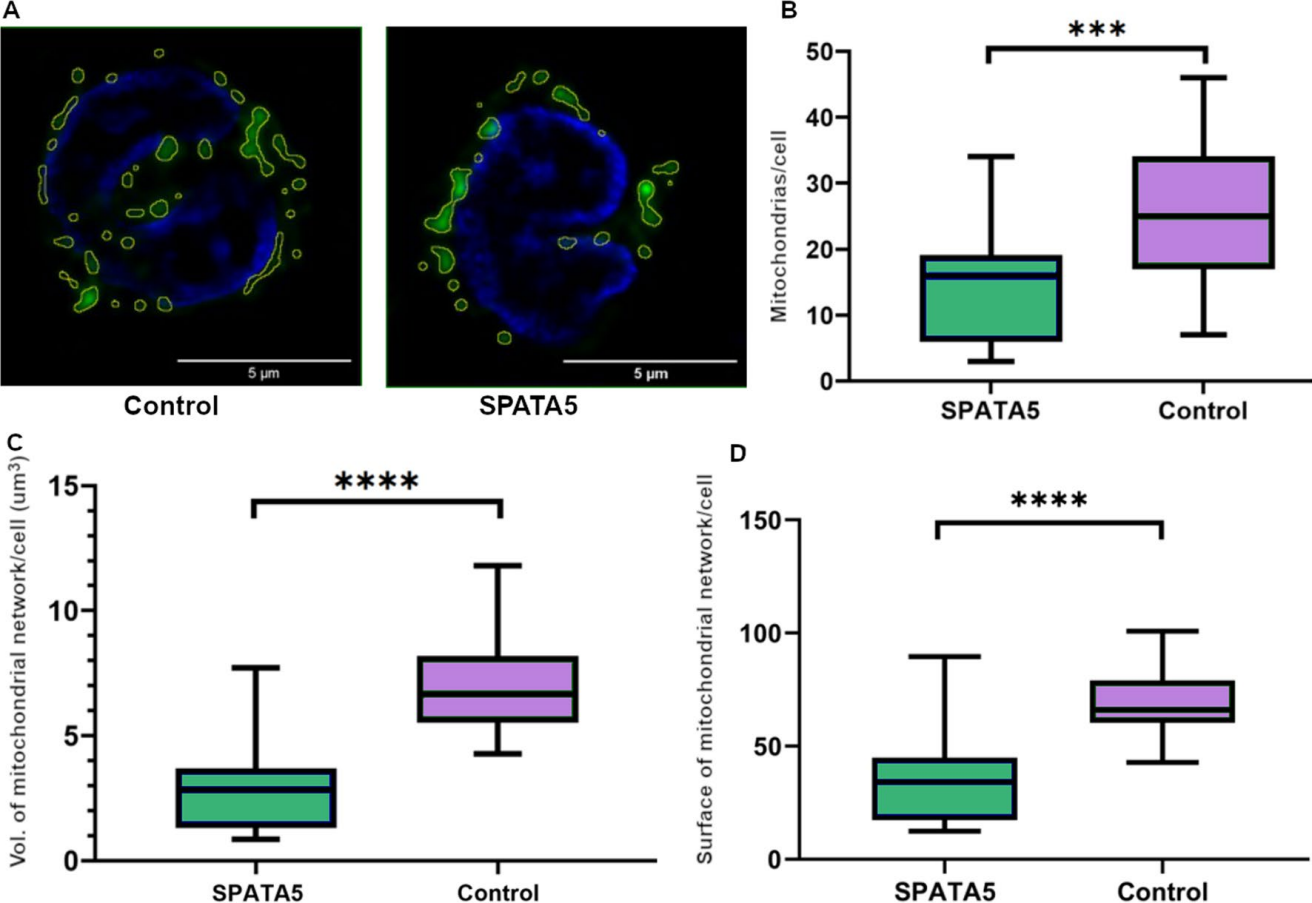
Monocyte mitochondrial mass. **A** Representative confocal image showing mitochondria (green) and nuclei (blue) stained with MitoTracker Green and DAPI, respectively. The regions of interest containing the mitochondria analyzed are marked in yellow. **B** Quantity of mitochondria per monocyte in case (*SPATA5*) compared to a healthy control (Control). **C** Total mitochondrial volume per monocyte in case (*SPATA5*) compared to a healthy control. **D** Mitochondrial total surface per monocyte in case (*SPATA5*) compared to a healthy control. (Unpaired t tests were performed, ***p < 0.001, ****p < 0.0001 (*n* = 15–27)

We analyzed the mitochondria from 15 monocytes from the patient and 27 from a healthy control (a 20-year-old female), after staining live cells with MitoTracker green, considering following morphological parameters: mitochondrial number, total mitochondrial volume, and total mitochondrial surface (Fig. 3). Figure 3A shows a representative confocal image of the mitochondrial network and cell nuclei. Mitochondrial quantity was on average 14 ± 2 mitochondria per cell in the patient and 25 ± 2 mitochondria per cell in the control (Fig. 3B). Mitochondrial total volume was on average 2.8 ± 0.5 µm^3^ in the patient and 6.9 ± 0.4 µm^3^ in the control (Fig. 3C). Total mitochondrial surface was 35 ± 5 µm^2^ on average in the patient and 70 ± 3 µm^2^ in the control (Fig. 3D). All in all, the three parameters measured were statistically different in control and patient (*p* value < 0.05). These results show an overall decrease in mitochondrial mass in patient’s monocytes with respect to the control group, and suggest an alteration in mitochondrial biogenesis or autophagy that has not been reported.

We also assessed mitochondrial morphology but did not find significant differences in the mitochondrial network between the patient and control subject (Additional file 2: Fig. S2).

### Mitochondrial function is impaired in patient’s platelets

Initially, mitochondrial function was assessed by analyzing the oxygen consumption rate in platelets and PBMCs from the patient and two healthy controls (18- and 20-year-old females).

**Fig. 4:**
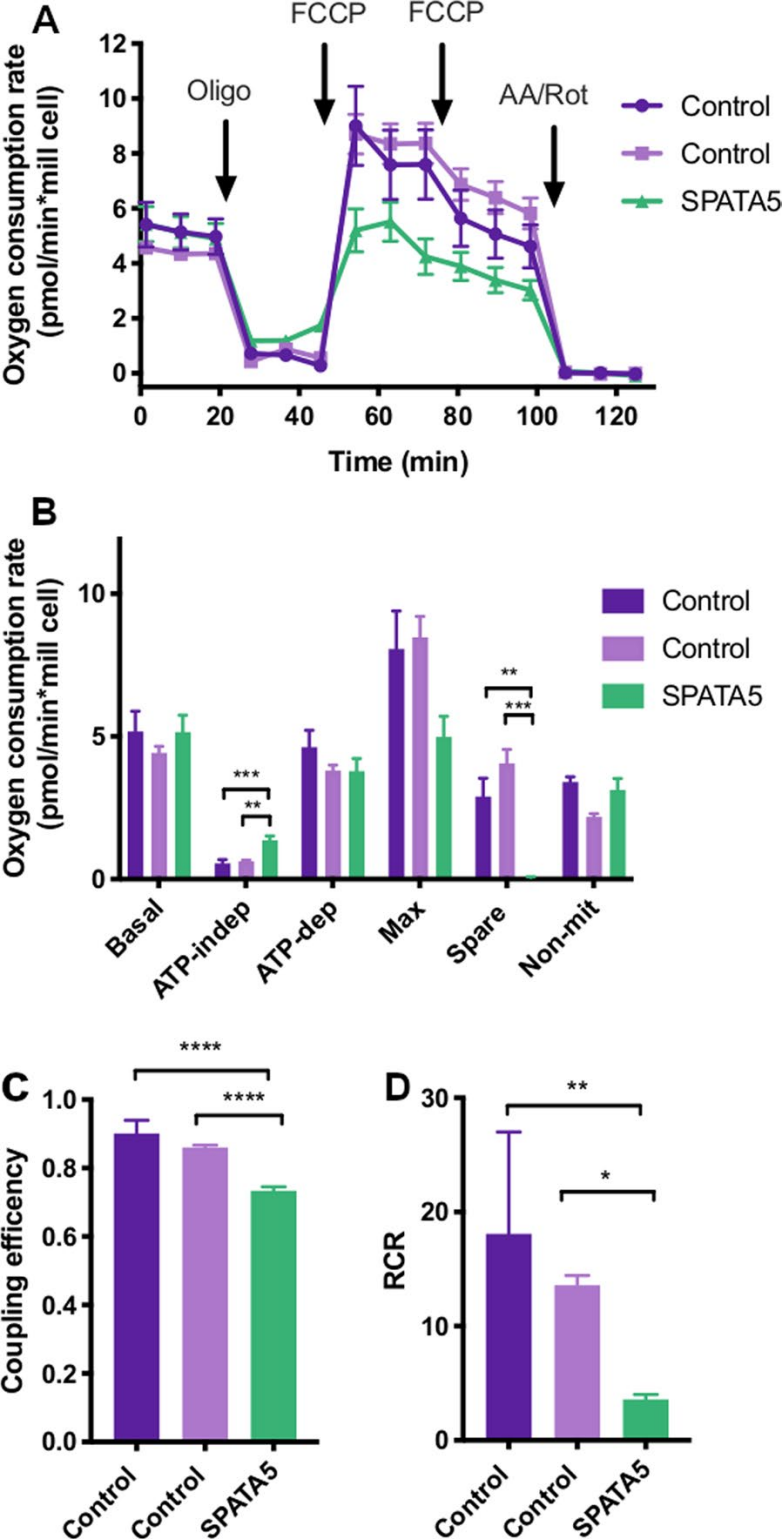
Platelet oxygen consumption rates. **A** Representative profiles of oxygen consumption rate (OCR) of platelets (2.5 × 10^7^ cells/well) isolated from blood samples. OCR was measured in a Seahorse XFe24 extracellular flux analyzer before and after the sequential addition of oligomycin (Oligo, final concentration 2.5 μM), FCCP (final concentrations 0.5 and 1 μM) and antimycin A plus rotenone (AA/Rot, final concentrations 2.5 μM/2.5 μM). All data were normalized to cell number. **B** Respiratory parameters and non-mitochondrial oxygen consumption rate were determined from the graph described above. **C** Coupling efficiency was the ratio between ATP-dependent and basal respiration rates. **D** Respiratory control ratio (RCR) was calculated as the ratio between maximum and ATP-independent respiration. Values are shown for the patient (SPATA5, green), female control subjects, 18 (dark purple) and 20 years old (light purple). Results are the mean ± SD. One-way ANOVA and Tukey post hoc tests were performed, *p < 0.05, **p < 0.01, ***p < 0.0001

Oxygen consumption rates of platelets from the patient and two young control subjects are shown in Fig. 4A. Several respiratory parameters were altered in the patient's platelets compared to the controls (Fig. 4B). The spare respiratory capacity was significantly reduced in the patient with respect to the controls, and the maximum respiration rate was also lower in the patients but not statistically significant. The maximum respiration rate and spare respiratory capacity usually correlate with the number of mitochondria and/or the activity of the electron transport chain complexes. These results are in agreement with the low respiratory complexes activities previously observed in patients with variants in the *SPATA* gene [5, 6].

Besides, the ATP-independent oxygen consumption rate was significantly higher in patients than control cells (Fig. 4B), suggesting an increase in proton leak. Coupling efficiency, determined as the ratio between ATP-dependent respiration and basal respiration, was lower in patient samples (Fig. 4C), indicating a likely impact of the mutations on ATP synthesis. Last, a decrease in respiratory control ratio (RCR), the ratio between maximum and ATP-independent respiration rates, was found in patient platelets compared to control subjects, supporting an overall decrease in mitochondrial function.

In PBMCs, most of the respiratory parameters remained unchanged, but a significant increase in non-mitochondrial oxygen consumption rate was observed in the patient cells with respect to the controls (Additional file 3: Fig. S3), in agreement with our previous report [7]. Non-mitochondrial respiration is usually linked to an increase in the activity of oxygen-consuming enzymes (such as NAD(P)H oxidases) or other processes that can produce oxidant species [17]. In these cells, we also assessed mtDNA status and found 300 copies of mtDNA per cell in the patient, lower than the values present in control samples (560 and 420 copies of mtDNA/cell in Control #1 and #2, respectively) in agreement with lower mitochondrial mass.

**Fig. 5:**
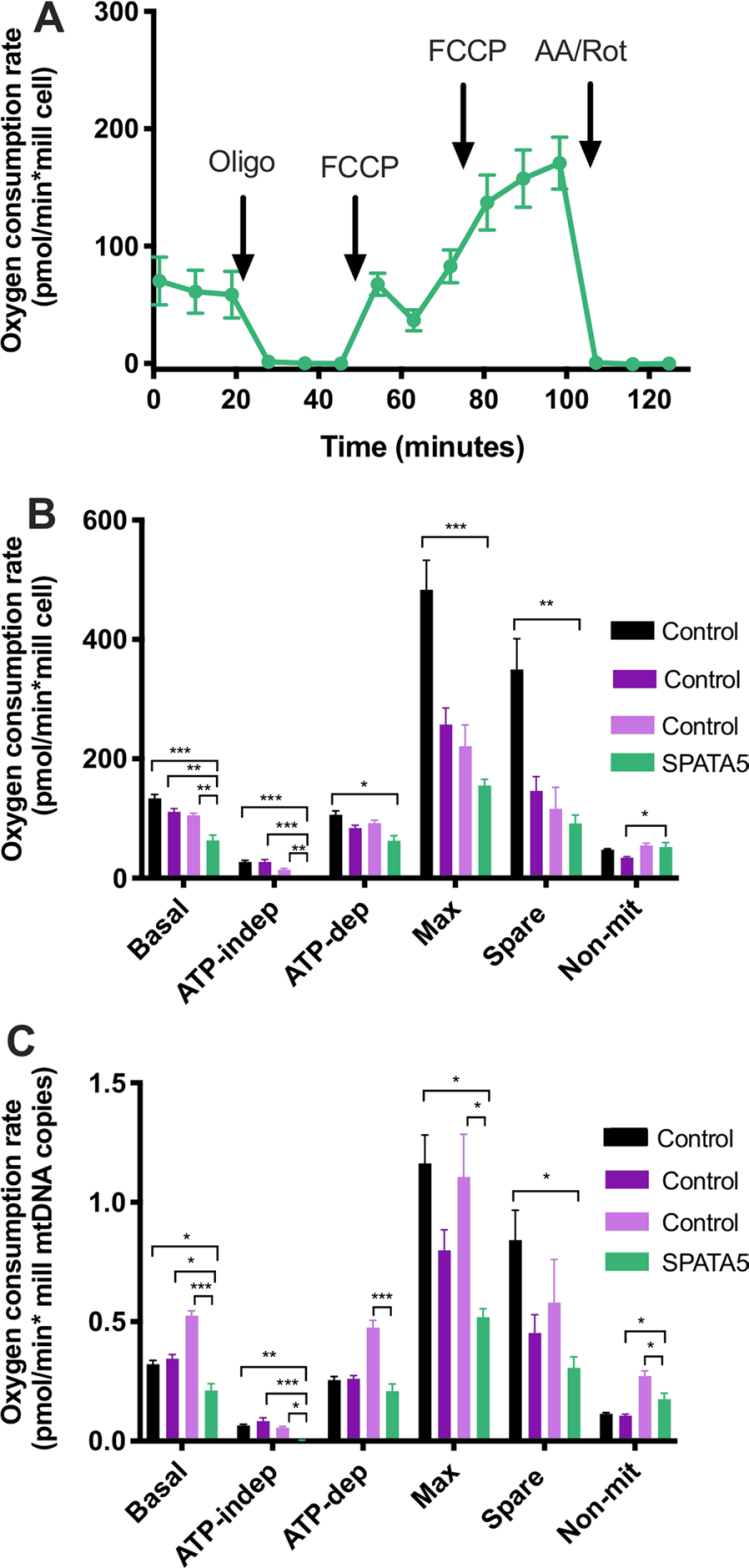
PBMC oxygen consumption rates. **A** Representative profile of oxygen consumption rate (OCR) of PBMCs (4 × 105 cells/well) isolated from a blood sample of the patient. OCR was measured in a Seahorse XFe24 extracellular flux analyzer before and after the sequential addition of oligomycin (Oligo, final concentration 2.5 μM), FCCP (final concentrations 1 and 3 μM) and antimycin A plus rotenone (AA/Rot, final concentrations 2.5 μM/2.5 μM). All data were normalized to cell number. **B** Respiratory parameters for the patient were determined from the graph described above. Control values were obtained from male pediatric subjects with 5, 7 and 10 years, the mean values of these subjects were published previously [33]. **C** Respiratory parameters were normalized considering the number of mtDNA copies of the patient (299 mtDNA copies/cell), and control subjects (416, 322 and 200 mtDNA copies/cell, for the 5, 7 and 10 years old male controls, respectively). Values are shown for the patient (SPATA5, green), male control subjects of 5 (Black), 7 (dark purple) and 10 years old (light purple). Results are the mean ± SD. One-way ANOVA and Dunnett’s post hoc tests were performed (*n* = 5), *p < 0.05, **p < 0.001, ***p < 0.0001

Since respiratory rates of PBMCs decay with age [33] and are reported to be higher in females than males [34], we compared the oxygen consumption rate of the patient with those of three healthy control male patients (5, 7 and 10 years old) from a recent study published by our group [33]. As shown in Fig. 5, basal respiration was significantly lower in the patient than in all control subjects, both when oxygen consumption rate was normalized by cells or by mtDNA copies. A decrease was also found in ATP-dependent and maximum respiration, as well as in the respiratory capacity of the patient with respect to controls, and statistical significance was achieved in many cases. These values strongly suggest an impairment in mitochondrial function in the patient.

## Discussion

In this study, we evaluated a patient with a rare and severe neurological disease of suspected genetic etiology via whole genome sequencing and found two novel variants in *trans* configuration in the *SPATA5* gene. Evidence for considering these two variants as causative is as follows. According to ACMG variant interpretation guidelines [35], we consider the frameshift variant fulfills the following pathogenicity criteria: (i) PVS1, since *SPATA5* is predicted to have a LOF mechanism of disease by NMD according to in silico predictions in this study and others [2]; (ii) PM2, since no control population contains the variant according to several genomic studies, including ExAC [36], Gnomad [37] and Kaviar [38]; (iii) PM3, since the variant was detected in trans with the other missense (likely pathogenic) variant (sanger sequencing of parents confirmed the phase); and (iv) PP4, since the patient’s phenotype is highly specific for a disease with a single genetic etiology. Hence, PVS1, two PM and one PP are sufficient evidence for a “pathogenic” classification for this variant.

The missense variant fulfills at least, (i) PM2 (low population frequency), (ii) PM3, same rationale as before, (iii) PP2 (missense variant in a gene that has a low rate of benign missense variation and where missense variants are a common mechanism of disease), (iv) PP3, since PolyPhen [39], Sift [40, 41], Mutation Taster [42], FATHMM [43] and LRT [44] have deleterious predictions and phyloP scores (placental and vertebrate, meaning that only placentates or vertebrates were used to construct the alignments for the scores) [40] are greater than 1.6 (2.643, 8.738 respectively), hence it is a conserved region, suggesting pathogenicity, (v) PP4, same rationale as above. Hence, two PM and two or more PP are enough evidence for a “likely pathogenic” classification. However, we believe that we could add some evidence for PS3 (which would change the classification to “pathogenic”), since we have performed well-established in vitro functional studies supportive of a damaging effect on the gene product. In addition, there are some elements to believe PM1 could also apply, since the mutation is in the most functionally relevant domain of the protein (AAA2 domain).

From the ENCODE project, we obtained RNA-seq from different tissues, including skeletal muscle and spinal cord. Both of them could be relevant for the phenotype under study. Expression signal is observed all along the *SPATA 5* gene, especially reads signal is observed toward the end of the transcripts supporting the expression of longer *SPATA5* isoforms (Additional file 4: Fig. S4). Those transcripts are harboring both mutations.

We believe these variants are causal of the phenotype, based on clinical presentation being compatible with the phenotypes described for variants in this gene, various in silico analysis and mitochondrial function studies.

A recent report [5, 6] described several patients with heterozygous variants in the *SPATA5* gene that were clinically suspected to present a mitochondrial disease, one of which presented defects in complex I and V in muscle. In this work, the authors also silenced *SPATA5* expression in rat cortical neurons and observed altered mitochondrial dynamics, a decrease in mitochondrial length, as well as in the ATP/ADP ratio [5]. Another report [6] showed alterations in the levels of several mitochondrial proteins, as well as low enzymatic activity of respiratory complexes in a muscle biopsy of a patient carrying two pathogenic, compound heterozygous variants in the *SPATA5* gene. Accordingly, we evaluated some parameters of mitochondrial physiology in blood cells from the patient and young control individuals. Our results showed an overall lower mitochondrial mass in monocytes obtained from the patient, compared to the control individual (Fig. 3). While the control is a young female, the patient is a male and some reports have shown that differences between sexes in mitochondrial mass could exist [34, 45, 46], though these appear to depend on the tissue and species. However, age-matched healthy pediatric controls are difficult to include in our studies, and a recent report shows a lack of significant differences in citrate synthase levels or mtDNA copies (classical markers of mitochondrial mass) in monocytes from men and women [47], supporting the comparison presented herein.

In platelets, oxygen consumption rate analysis revealed a significant increase in ATP-independent respiration, also known as “leak”, and decrease of the spare respiratory capacity in platelets from the patient, with respect to the healthy controls (Fig. 4). Respiratory indices were calculated and significantly lower coupling efficiency and respiratory control rate were found in platelets of the patient with respect to healthy controls (Fig. 4). The controls were two young females; however, previous reports show that sex and age do not affect platelet respiration [33, 48], supporting the validity of our results.

We also measured oxygen consumption rates in PBMCs. However, we did not find significant differences in mitochondrial respiratory parameters between the patient and female control individuals 18 and 20 years old, (Additional file 3: Fig. S3); only an increase in non-mitochondrial respiration. Since PBMCs respiration rates are reported to be higher in females than males [34], and to decrease with age [7], we compared the patient’s respiratory parameters with those of age-matched male controls from a recent study published by our group [7]. Several parameters indicative of mitochondrial function were significantly lower in the patient than in these controls, including basal respiration, maximum respiration and spare respiratory capacity. These results are similar to our previous observations in patients with mitochondrial disease, carrying mutations in mtDNA and nDNA [7].

We wondered if compensatory processes, such as an increase in mitochondrial biogenesis, could be taking place in PBMCs [7, 49–52]. To explore this possibility, we determined the number of copies of mtDNA in PBMCs, from the patient and control individuals, but found lower or similar levels in the patient. This result supports the decrease in mitochondrial mass, rather than the existence of a compensatory biogenesis program, in the patient. Nevertheless, other compensatory events such as an increase in catabolic pathways that provide substrates for the electron transport chain, cannot be discarded [53].

. In a recent report, we presented oxygen consumption rates for platelets and PBMCs from young control subjects and patients with mitochondrial disease, confirmed at the molecular level [7]. Interestingly, the values of most respiratory parameters (basal, ATP-dependent, maximum respiration and spare respiratory capacity) in platelets and PBMCs from our patient, carrying the variants in *SPATA5,* were similar or lower than the median values observed for the group of patients analyzed previously [7]. While the ATP-independent respiration in platelets (that denotes uncoupling), and the non-mitochondrial respiration (associated with reactive oxygen species) in both platelets and PBMCs from the *SPATA5* patient were higher than those observed in our previous studies [7]. These results further support the value of studying mitochondrial function in blood cells for the diagnosis of mitochondrial disease. In sum, our observations suggest the *SPATA5* mutation affects mitochondrial ATP synthesis, as well as the ability to respond to an increase in energy demand, in both platelets and PBMCs, probably due to a decrease in mitochondrial mass [17]. The results are in agreement with previous observations [5, 6] and constitute the first report of impaired mitochondrial mass and respiration in cells derived from a patient carrying mutations in the SPATA5 gene.

**Fig. 6:**
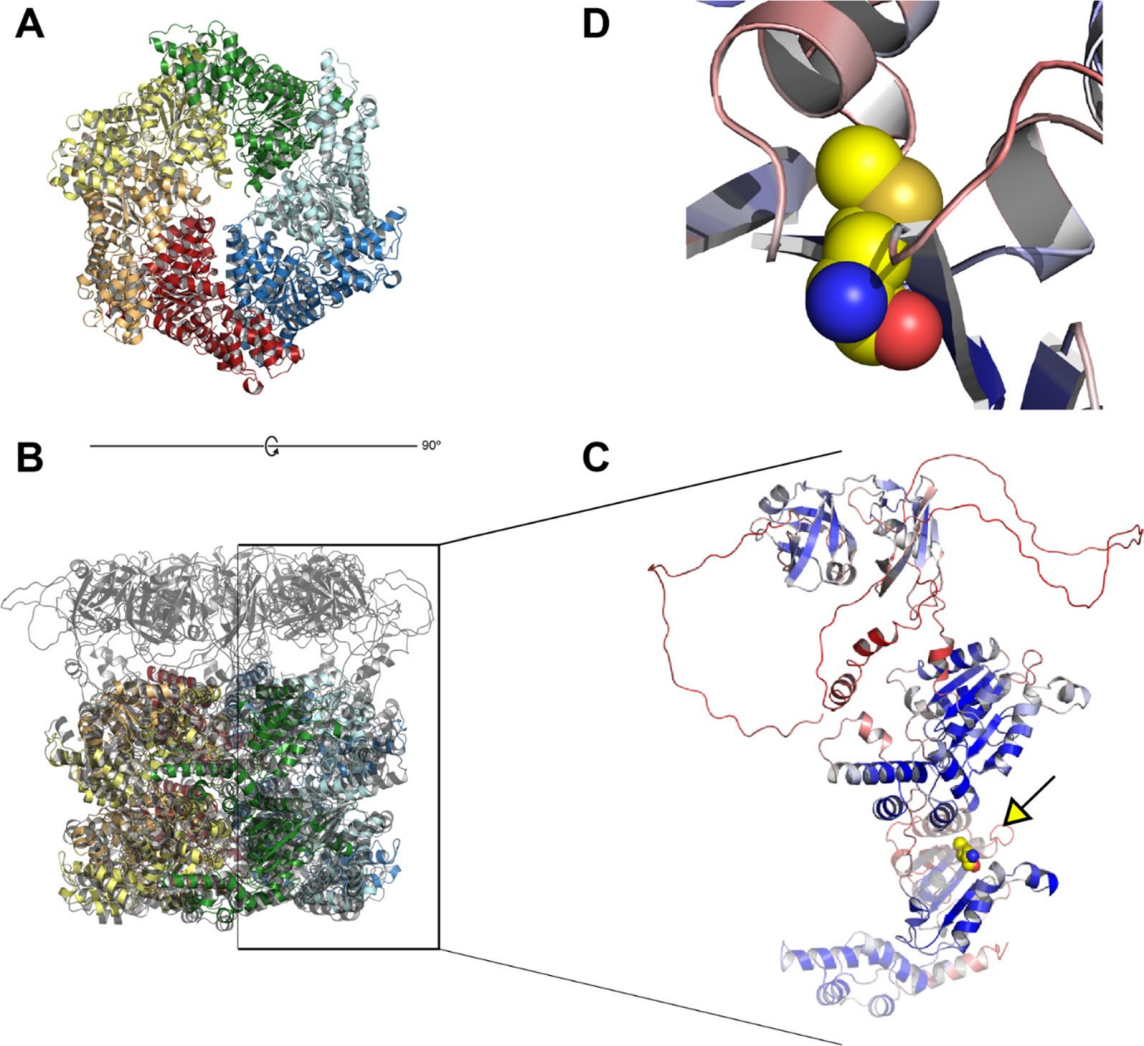
Protein structure modeling and comparative analyses of SPATA5 protein (ATPase family protein 2 homolog). **A** “Bottom” view of the hexameric experimental structure of *SPATA5* homolog in *S. cerevisiae* (PDB 7KNU), colored by chain. **B** Rotated yeast hexamer superposed to model for *SPATA5* homohexamer, manually built with AlphaFold monomers, in transparent gray cartoon. **C** Detail of the AlphaFold model for V766M *SPATA5*, color-coded from very high confidence (pLDDT > 90, dark blue) to very low confidence (pLDDT < 50, dark red). The C-terminal portion (Ile 677→end) affected by the frameshift mutation is shown as a transparent cartoon, coinciding with the very beginning of the second AAA + domain. The arrow depicts the connector affected by the V766M, represented as spheres. **D** Met766 substitutes a strictly conserved valine, affecting the beta 3 alpha2 connection composing the alpha/beta architecture of the AAA + domain (see “Discussion” Section)

Structural characterization of AFG2, *SPATA5*’s homolog from baker’s yeast has shown it exists as a homohexamer [54]. Though some 100 residues shorter, AFG2 is reliably alignable to *SPATA5* over its whole length. The reliable portion of a *SPATA5* structural model obtained with AlphaFold [26], showed it to be superposable to the hexamer-forming domains of AFG2 (Fig. 6b–d). The poorly modeled N-terminal region (up to residue 350) reflects limited available sequences to train the deep-learning method (Fig. 5c). Indeed, this ~ 350 residue region looks confined to mammals, with very conserved sequence positions throughout the group. The six N-terminal regions of *SPATA5* would be displaying a rich region for protein–protein interactions out of the hexamer. Three points support this hypothesis: (i) AF models with no assigned secondary structure often coincide with disordered or coil regions; (ii) a reliable portion of the N-terminal model is reminiscent of small beta barrel domains used as protein–protein interaction modules, e.g., as observed in Pleckstrin-homology domains [55]; (iii) finally, *SPATA5* is thought to be a hub of protein interactions, engaging in many productive contacts [5].

We think that this finding can contribute to the use of whole genome sequencing as a diagnostic tool for challenging rare diseases, especially when no etiological orientation is given. Additionally, this kind of data might enhance the set of known mutations associated with different diseases. We think sharing this information is crucial for pushing genomic medicine further and improving diagnostic yields.

## Supplementary Information

**Additional file 1. Table S1.** Primers targeting a region of the nuclear gene β2M and the minor arc of mtDNA.

**Additional file2**. **Figure S2**: Mitochondrial morphology in monocytes. A. Representative confocal fluorescence image showing mitochondria (green) and nuclei (blue) stained with MitoTracker Green and DAPI, respectively, of a monocyte from the patient. The regions of interest containing the skeleton images of mitochondria analyzed are marked in different colors depending on the shape of the mitochondria: blue (puncta), orange (filament) and purple (network). B. Quantity of mitochondria per monocyte with each of the morphologies analyzed, obtained from skeletonized images, of the case (SPATA5) compared to a healthy control (Control, female 20 year old). Mann–Whitney test was applied (n=15-27), NS= nonsignificant.

**Additional file3**. **Figure S3**: PBMC oxygen consumption rates. A. Representative profiles of oxygen consumption rate (OCR) of PBMCs (4 x 105 cells/well) isolated from blood samples. OCR was measured in a Seahorse XFe24 extracellular flux analyzer before and after the sequential addition of oligomycin (Oligo, final concentration 2.5 μM), FCCP (final concentrations 1 and 3 μM) and antimycin A plus rotenone (AA/Rot, final concentrations 2.5 μM/2.5 μM). All data were normalized to cell number. B. Respiratory parameters and non-mitochondrial oxygen consumption rate were determined from the graph described above. Values are shown for the patient (SPATA5, green), female control subjects of 18 (dark purple) and 20 years old (light purple).Results are the mean ± SEM. One-way ANOVA and Tukey post hoc tests were performed (n = 5), *p<0.05.

**Additional file4**. **Figure S4**: RNA-seq expression data obtained from the ENCODE project. Skeletal muscle and spinal cord tissue are observed. Read signal is observed all along the SPATA5 gene, not restricted to short isoforms.

## Data Availability

Data regarding both genomic variants is available in ClinVar under records VCV001679513.1 and VCV001679511.1?
