# Supplementary figures and images for: Computational and mitochondrial functional studies of novel compound heterozygous variants in SPATA5 gene support a causal link with epileptogenic encephalopathy
Source: Hum Genomics. 2023 Feb 27;17:14. doi: 10.1186/s40246-023-00463-x (PMC9972848; doi:10.1186/s40246-023-00463-x)

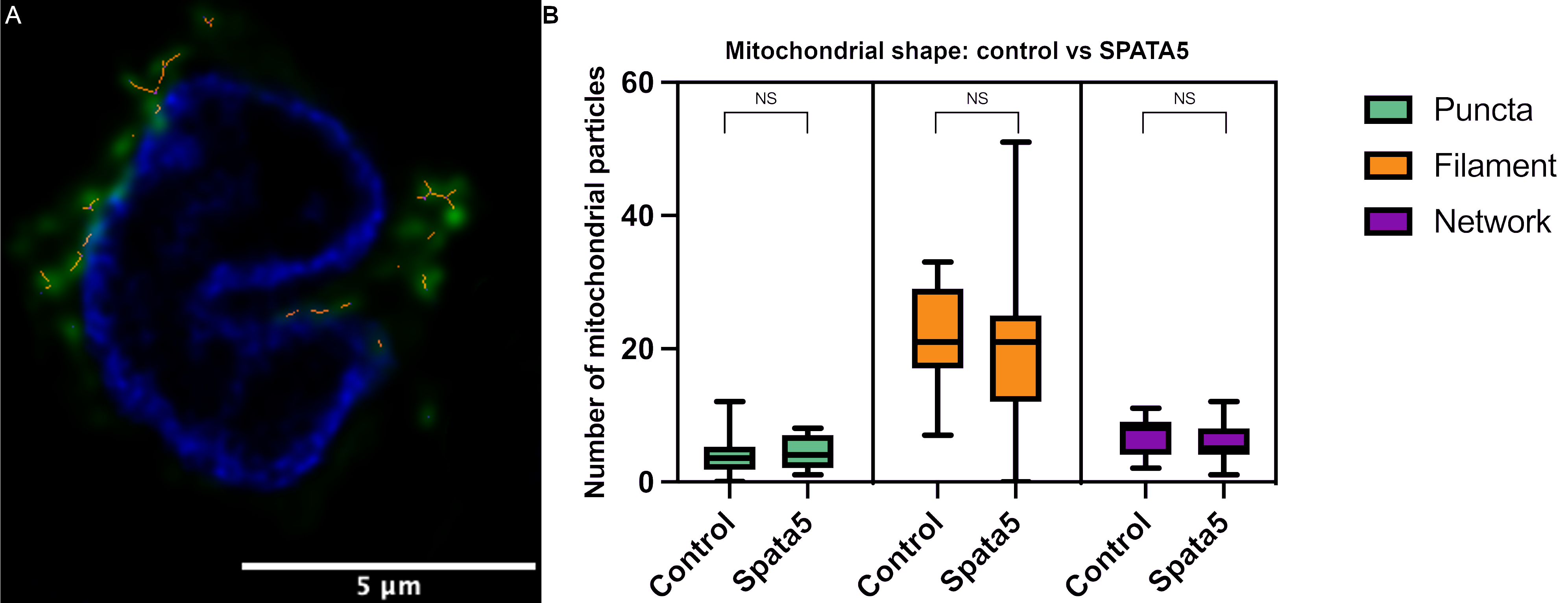

Supplement: Supplementary file 2 — Additional file2. Figure S2: Mitochondrial morphology in monocytes. A. Representative confocal fluorescence image showing mitochondria (green) and nuclei (blue) stained with MitoTracker Green and DAPI, respectively, of a monocyte from the patient. The regions of interest containing the skeleton images of mitochondria analyzed are marked in different colors depending on the shape of the mitochondria: blue (puncta), orange (filament) and purple (network). B. Quantity of mitochondria per monocyte with each of the morphologies analyzed, obtained from skeletonized images, of the case (SPATA5) compared to a healthy control (Control, female 20 year old). Mann–Whitney test was applied (n=15-27), NS= nonsignificant. [file 40246_2023_463_MOESM2_ESM.tif]

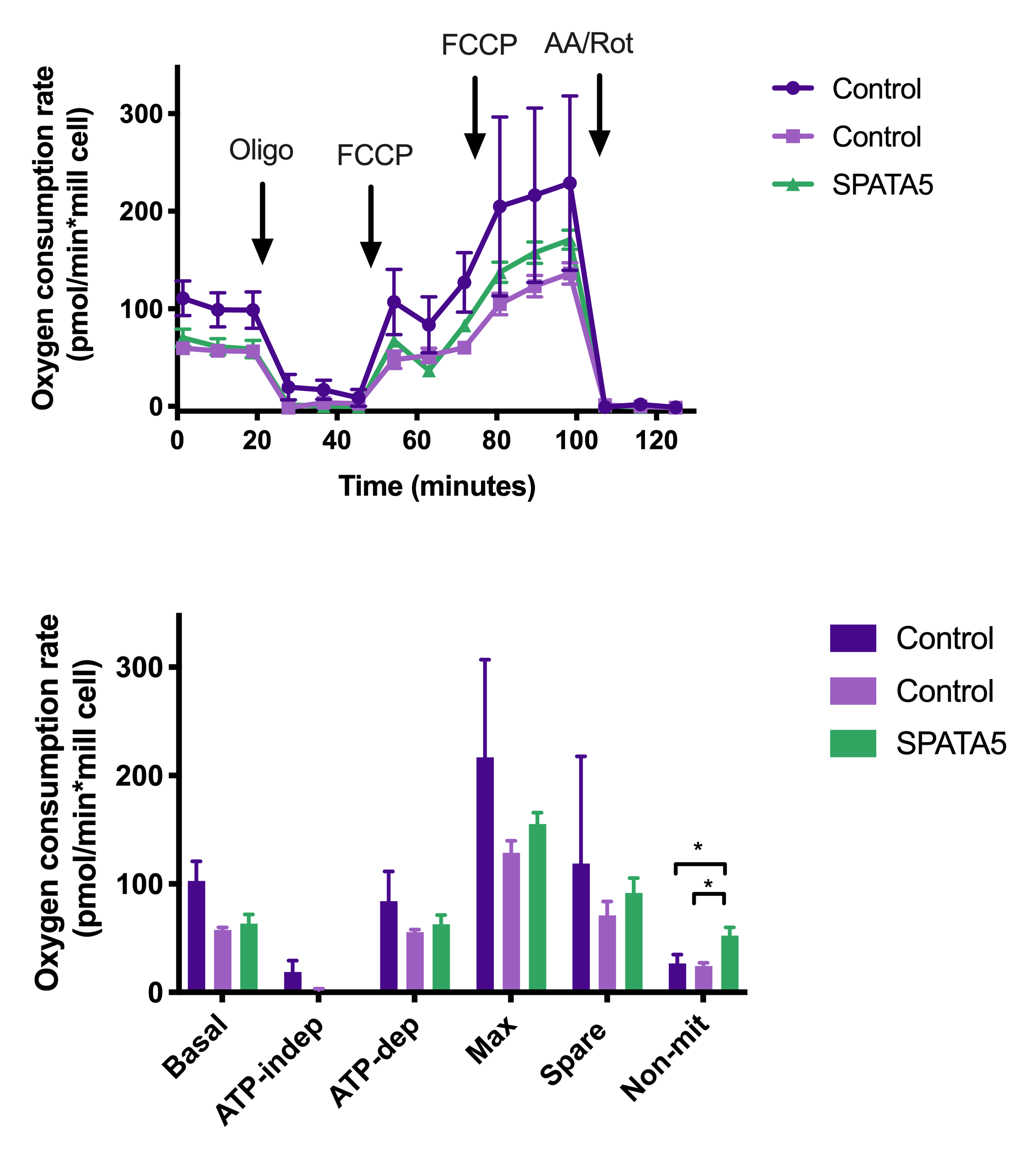

Supplement: Supplementary file 3 — Additional file3. Figure S3: PBMC oxygen consumption rates. A. Representative profiles of oxygen consumption rate (OCR) of PBMCs (4 x 10^5^ cells/well) isolated from blood samples. OCR was measured in a Seahorse XFe24 extracellular flux analyzer before and after the sequential addition of oligomycin (Oligo, final concentration 2.5 μM), FCCP (final concentrations 1 and 3 μM) and antimycin A plus rotenone (AA/Rot, final concentrations 2.5 μM/2.5 μM). All data were normalized to cell number. B. Respiratory parameters and non-mitochondrial oxygen consumption rate were determined from the graph described above. Values are shown for the patient (SPATA5, green), female control subjects of 18 (dark purple) and 20 years old (light purple).Results are the mean ± SEM. One-way ANOVA and Tukey post hoc tests were performed (n = 5), *p<0.05. [file 40246_2023_463_MOESM3_ESM.tiff]

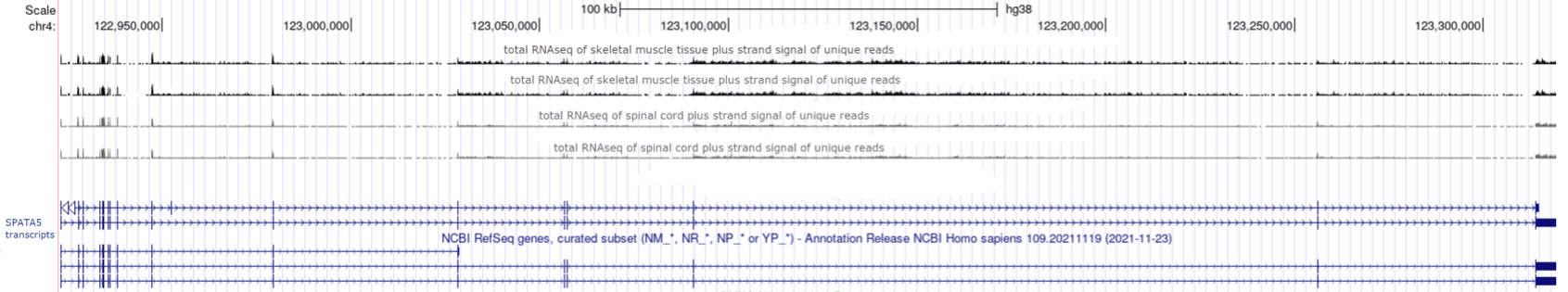

Supplement: Supplementary file 4 — Additional file4. Figure S4: RNA-seq expression data obtained from the ENCODE project. Skeletal muscle and spinal cord tissue are observed. Read signal is observed all along the SPATA5 gene, not restricted to short isoforms. [file 40246_2023_463_MOESM4_ESM.tiff]
